# Improving the diagnostic process for patients with possible bladder and kidney cancer: a mixed-methods study to identify potential missed diagnostic opportunities

**DOI:** 10.3399/BJGP.2022.0602

**Published:** 2023-05-31

**Authors:** Yin Zhou, Hardeep Singh, Willie Hamilton, Stephanie Archer, Sapphire Tan, James Brimicombe, Georgios Lyratzopoulos, Fiona M Walter

**Affiliations:** Primary Care Unit, Department of Public Health and Primary Care, University of Cambridge, Cambridge, UK.; Center for Innovations in Quality, Effectiveness and Safety, Michael E DeBakey Veterans Affairs Medical Center and Baylor College of Medicine, Houston, TX, US.; University of Exeter Medical School, Exeter, UK.; Department of Public Health and Primary Care, University of Cambridge, Cambridge and Department of Psychology, University of Cambridge, Cambridge, UK.; Primary Care Unit, Department of Public Health and Primary Care, University of Cambridge, Cambridge, UK.; Primary Care Unit, Department of Public Health and Primary Care, University of Cambridge, Cambridge, UK.; Epidemiology of Cancer Healthcare and Outcomes (ECHO), Department of Behavioural Science and Health, Institute of Epidemiology and Health Care (IEHC), University College London, London, UK.; Department of Public Health and Primary Care, University of Cambridge, Cambridge and Wolfson Institute of Population Health, Queen Mary University of London, London, UK.

**Keywords:** bladder cancer, early diagnosis of cancer, kidney cancer, missed opportunities, primary health care

## Abstract

**Background:**

Patients with bladder and kidney cancer may experience diagnostic delays.

**Aim:**

To identify patterns of suboptimal care and contributors of potential missed diagnostic opportunities (MDOs).

**Design and setting:**

Prospective, mixed-methods study recruiting participants from nine general practices in Eastern England between June 2018 and October 2019.

**Method:**

Patients with possible bladder and kidney cancer were identified using eligibility criteria based on National Institute for Health and Care Excellence (NICE) guidelines for suspected cancer. Primary care records were reviewed at recruitment and at 1 year for data on symptoms, tests, referrals, and diagnosis. Referral predictors were examined using logistic regression. Semi-structured interviews were undertaken with 15 patients to explore their experiences of the diagnostic process, and these were analysed thematically.

**Results:**

Participants (*n* = 940) were mostly female (*n* = 657, 69.9%), with a median age of 71 years (interquartile range 64–77 years). In total, 268 (28.5%) received a referral and 465 (48.5%) had a final diagnosis of urinary tract infection (UTI). There were 33 (3.5%) patients who were diagnosed with cancer, including prostate (*n* = 17), bladder (*n* = 7), and upper urothelial tract (*n* = 1) cancers. Among referred patients, those who had a final diagnosis of UTI had the longest time to referral (median 81.5 days). Only one-third of patients with recurrent UTIs were referred despite meeting NICE referral guidelines. Qualitative findings revealed barriers during the diagnostic process, including inadequate clinical examination, female patients given repeated antibiotics without clinical reviews, and suboptimal communication of test results to patients.

**Conclusion:**

Older females with UTIs might be at increased risk of MDOs for cancer. Targeting barriers during the initial diagnostic assessment and follow-up might improve quality of diagnosis.

## INTRODUCTION

Most patients with bladder and kidney cancer in the UK are diagnosed following a referral from a GP.^1^ Initial blood and urine tests are usually performed in primary care following symptomatic presentation, with referrals initiated if further imaging or specialist input is required. Patients with bladder and kidney cancer with prior benign conditions such as urinary tract infections (UTIs) commonly experience diagnostic delay,^2^^,^^3^ and patients with recurrent UTIs are less likely to receive guideline concordant care.^4^ Given that urine culture results take a few days to receive, many UTIs diagnosed in primary care are presumptive. These unconfirmed episodes can be considered as ‘presumed UTIs’.

Retrospective cohort studies demonstrate that signals of missed opportunities exist in patients diagnosed with cancer,^5^ including those with bladder and kidney cancer.^4^^,^^6^^,^^7^ The concept of missed diagnostic opportunities (MDOs) represents situations where, in retrospect, something different could have been done to achieve a more timely diagnosis in the context of an evolving diagnostic process.^5^ Delayed diagnosis is associated with poorer patient experience^8^^,^^9^ and health outcomes.^10^ Nevertheless, when and how suboptimal care might occur during the diagnostic process of bladder and kidney cancer is largely unknown.

In this prospective study, titled ‘Urological symptoms and pathways to diagnosis (USP)’, two conceptual frameworks were used to guide the methods and analysis:

- the pathways to treatment model; and
- ‘*Safer Dx*’.

The pathways to treatment model describes measurable time points and intervals during the diagnostic pathway of patients with cancer and the contributory factors that might affect each stage of the pathway.^11^ This study mostly focused on the primary care interval (PCI, from patient presentation to referral), and the overall diagnostic interval (from patient presentation to diagnosis).

The second conceptual framework, *Safer Dx*, is used in diagnostic safety research to characterise potential contributory factors to breakdowns in the processes of care relating to diagnosis.^12^ This model was used in the current study to frame the mixed-methods analysis and interpretation of the findings. The overall study aim was to examine characteristics of potential MDOs in patients at increased risk of bladder and kidney cancer, so that targeted interventions can be developed to improve the quality of the diagnostic process for these patients.

**Table:** How this fits in

Currently there is variation in diagnostic timeliness among patients with bladder and kidney cancer. How and why suboptimal care occurs is unknown. The current study found that patients with presumed urinary tract infections (UTIs) were at increased risk of experiencing potential missed diagnostic opportunities (MDOs). There was evidence of potential MDOs including inadequate clinical examination, repeated antibiotic prescribing without reviews or referrals, and lack of or poor communication of test results resulting in no appropriate follow up. Improving clinician awareness of at-risk groups, tightening guideline recommendations on management of UTIs, and improving practice communication of test results might help to reduce MDOs in patients with possible bladder and kidney cancer.

## METHOD

### Research design

The USP study was a mixed-methods prospective study consisting of case-note reviews and nested interviews. Patients were recruited between June 2018 and October 2019 from nine primary care (GP) practices with a list size >10 000 in Eastern England (see Supplementary Table S1). The authors chose to focus on patients with National Institute for Health and Care Excellence (NICE) relevant symptoms for possible bladder and kidney cancer^13^ and large practices to maximise the chances of identifying patients with cancers. The primary care practices were recruited via the Eastern Clinical Research Network (CRN).

### Patient recruitment

First an initial electronic (e-)search was performed dating back 3 months before the search date in each practice. Subsequently, regular 2–4 weekly e-searches were undertaken to identify patients who had presented with relevant symptoms listed in the eligibility criteria (Table 1). These features have positive predictive values of >3%, either based on their inclusion in the 2015 NICE guidelines for suspected cancer^13^ or from the broader literature.^14^^,^^15^ E-searches were developed by two CRN information technology specialists for the two primary care clinical systems used by the recruited practices, SystmOne and EMIS, and piloted in five general practices to ensure their validity. A clinical researcher (research nurse or GP) reviewed each identified patient's records to determine inclusion and exclusion criteria for eligibility.

**Table 1.** Inclusion and exclusion criteria for the USP study

**Criteria**
**Inclusion** Patients with the following symptoms/signs listed in the National Institute for Health and Care Excellence 2015^13^ guidelines for suspected cancer, or the broader literature: Aged >45 years, macroscopic or microscopic haematuria Aged >60 years, with a urinary tract infection (UTI), or any of the following symptoms suggestive of a UTI: dysuria urgency frequency nocturia incontinence Aged >60 years, abnormal urine dipstick: leucocytes or nitrites Aged >60 years, abdominal pain, low mean corpuscular volume

**Exclusion**
Already diagnosed kidney, bladder, or prostate cancer Deemed lacking capacity to provide informed consent by practice clinician

Eligible patients were posted an information pack comprising an invitation letter, patient information sheet, consent form, and Freepost reply envelope. Consent was sought for access to primary care records and record review, and an indication of their interest in a qualitative interview.

Following the initial search and invitation at each practice, a second e-search was performed concurrently with the first to identify consented participants who had been referred to secondary care for an abdominal or pelvic ultrasound or to a relevant specialty (emergency care, urology, gynaecology, gastroenterology, or colorectal). This was to maximise the chances of identifying patients who were subsequently diagnosed with cancer. Consented participants' records were also screened by the study team for referrals to identify any missed cases from the e-searches. Once the secondary care investigations were performed, the patient was contacted for their interest in a qualitative interview, and an interview information sheet and reply slip sent if they indicated interest. Informed consent was taken before the interviews. Supplementary Figure S1 shows the study procedures in a flow chart. As a result of the emerging themes from the initial interviews indicating the possibility of MDOs in patients with presumed UTIs, the second batch of interviews were carried out with patients with persistent or recurrent UTIs who did not receive any referrals.

### Data collection

Data were extracted from participants' notes by clinical researchers from the practices, CRN, or the research team (two GPs, three research nurses, and one medical student) at or soon after recruitment, and at 1 year following recruitment, using a Microsoft Access data collection application specially developed for the USP study. Information on patient demographics (age, gender, occupation, relevant family history, Index of Multiple Deprivation [IMD] based on postcode,^16^ body mass index [BMI], and smoking status), past medical history, drug history, index consultation, presenting symptoms, subsequent consultations, primary and secondary care tests, referrals, and final diagnosis were collected.

Interviewees were asked about their experiences of the diagnostic process relating to test use in primary and secondary care where appropriate. The first group of interviews with referred patients were undertaken in patients' homes between May and August 2019, and the second group of non-referred patients were interviewed by phone in May 2020 (because of the COVID-19 pandemic).

A practical decision was taken to conclude the qualitative study at 15 interviews owing to the impact of the COVID-19 pandemic on research activities in the country. Interviews were performed by two researchers using a semi-structured interview schedule. The interview schedule was developed and informed by literature, focusing on the patient, clinician, and healthcare factors that might contribute to MDOs. These factors included, but were not limited to, patient psychosocial issues (for example, fear of cancer or procedure and potential risks^17^^,^^18^), doctor–patient relationships (for example, continuity of care and trust), communication (of results in particular),^19^^–^^21^ and system access issues.

### Data preparation

The date of index consultation was the date of the first consultation that resulted in the patient being identified from the electronic search. This can be up to 3 months before the first search, or 2–4 weeks before subsequent searches. The final diagnosis was defined as one to which the symptoms at index consultation were attributed at the 12-month follow up. This may be a UTI confirmed during the index consultation (with no relevant subsequent consultations), or an eventual cancer diagnosis 3 months after the index consultation.

### Data analysis

Descriptive statistical analyses were conducted on symptoms, referral patterns, and diagnostic intervals (both PCI and overall diagnostic interval). In patients with multiple referrals, the first referral was considered to be the index referral, and PCI was calculated from the date of index consultation to date of the index referral. Covariates were chosen based on their data completeness and known prior risk factors for these cancers. Logistic regression was used to explore predictors of referral status (yes or no) by patient (gender, age, smoking status, IMD, and BMI) and clinical factors (presenting symptoms and final diagnosis), first crudely, then adjusted for all other independent variables. A sensitivity analysis was performed that included ‘final diagnosis’ as an additional independent variable in a third multivariable model. This was undertaken in case there was endogeneity between symptoms and final diagnosis recorded (that is, the two variables were not totally independent of each other).

Particular focus was given to patients with a recorded diagnosis or symptoms of UTI because of the authors' prior interest in their prolonged diagnostic intervals^4^ compared with other patient groups, and as they formed the largest symptomatic group in the current sample. Those with ≥3 episodes of UTIs in a 12-month period were regarded as ‘NICE qualifying’.^4^ UTI episodes in subsequent consultations after the index consultation were defined as a recording of any of the following:

- core UTI symptoms (dysuria, urgency, or frequency);
- UTI diagnosis given; or
- documented antibiotic given for UTI or its symptoms.

The PCI of the NICE-qualifying patient group was then examined. Qualitative interviews were recorded and transcribed verbatim. All transcripts were read numerous times by one researcher, and subsets of the data were reviewed by two other experienced qualitative researchers. The qualitative dataset was fine-coded and emergent themes identified using thematic analysis.^22^ Findings were discussed with the wider team that included primary care doctors, a psychologist, a public health consultant, and a professor of health services research and expert in diagnostic safety research, to reach an overarching understanding and interpretation of the findings.

## RESULTS

### Quantitative results

#### Participants

There were 2633 patients who were invited to join the study, with 974 (37.0%) patients consenting to participate. Ineligible patients were removed (pre-existing renal cancer [*n* = 1]; significant amount of missing data [*n* = 33]), so data from 940 participants were analysed. The participants were mostly female (*n* = 657, 69.9%) with a median age of 71 years (interquartile range 64–77 years) (see Supplementary Table S2).

Of the 775/940 (82.4%) patients with recorded symptoms, the frequency of reported symptoms at index consultation ranged between 41.0% for urinary frequency to 0.3% for anaemia (Table 2). The majority (*n* = 706, 75.1%) of patients reported up to three symptoms at index consultation (*n* = 255, 27.1% with one; *n* = 269, 28.6% with two; and *n* = 182, 19.4% with three symptoms) (data not shown).

**Table 2.** Logistic regression of predictors of having a referral

**Variables**	**Patients**		**Referred patients**		**Crude OR (95% CI)**	***P*-value^a^**	**Adjusted OR (95% CI)**	***P*-value** **^a^**

	***N***	**%**	***n***	**%**				
**Gender**								
Female	657	69.9	146	22.2	Reference	—	Reference	—
Male	283	30.1	122	43.1	2.65 (1.97 to 3.58)	<0.001	3.03 (2.12 to 4.34)	<0.001

**Age group, years**								
44–49	31	3.3	12	38.7	1.55 (0.70 to 3.45)	0.725	2.47 (1.01 to 6.04)	0.234
50–54	40	4.3	14	35.0	1.32 (0.63 to 2.76)	—	1.59 (0.70 to 3.60)	—
55–59	66	7.0	18	27.3	0.92 (0.49 to 1.75)	—	1.37 (0.68 to 2.75)	—
60–64	121	12.9	39	32.2	1.18 (0.71 to 1.98)	—	1.42 (0.81 to 2.50)	—
65–69	159	16.9	46	28.9	Reference	—	Reference	—
70–74	209	22.2	57	27.3	0.92 (0.58 to 1.46)	—	0.95 (0.58 to 1.56)	—
75–79	138	14.7	37	26.8	0.90 (0.54 to 1.50)	—	1.11 (0.64 to 1.92)	—
≥80	176	18.7	45	25.6	0.84 (0.52 to 1.37)	—	0.85 (0.50 to 1.44)	—

**Smoking status**								
Non-smoker	512	54.5	146	28.5	0.98 (0.72 to 1.34)	0.993	1.15 (0.81 to 1.63)	0.582
Smoker	66	7.0	18	22.7	Reference	—	Reference	—
Ex-smoker	308	32.8	89	28.9	0.92 (0.51 to 1.67)	—	1.01 (0.52 to 1.97)	—
Missing	54	5.7	15	27.8	0.95 (0.50 to 1.80)	—	1.64 (0.78 to 3.48)	—

**IMD quintile^b^**								
1 (least deprived)	190	20.2	70	36.8	2.03 (1.29 to 3.21)	0.004	1.94 (1.15 to 3.27)	0.044
2	184	19.6	50	27.3	1.30 (0.81 to 2.09)	—	1.52 (0.90 to 2.57)	—
3	203	21.6	67	33.0	1.72 (1.09 to 2.71)	—	1.66 (1.01 to 2.73)	—
4	171	18.2	38	22.2	1.00 (0.60 to 1.64)	—	1.03 (0.59 to 1.79)	—
5 (most deprived)	184	19.6	41	22.3	Reference	—	Reference	—

**BMI**								
<18.5	11	1.2	4	36.4	1.07 (0.30 to 3.82)	0.003	1.59 (0.38 to 6.64)	0.014
18.5–24.9	167	17.8	58	34.7	Reference	—	Reference	—
25–29.9	217	23.1	76	35.0	1.01 (0.66 to 1.55)	—	1.04 (0.65 to 1.65)	—
30–34.9	94	10.0	32	34.0	0.97 (0.57 to 1.65)	—	1.08 (0.61 to 1.92)	—
35–39.9	32	3.4	8	25.0	0.63 (0.26 to 1.48)	—	0.60 (0.24 to 1.55)	—
>40	20	2.1	6	30.0	0.81 (0.29 to 2.21)	—	0.86 (0.28 to 2.65)	—
Missing	399	42.4	84	21.1	0.50 (0.34 to 0.75)	—	0.52 (0.33 to 0.81)	—

**Presenting symptoms (yes versus no)**								
Urinary frequency	382	40.6	94	24.6	0.72 (0.54 to 0.97)	0.029	0.77 (0.55 to 1.10)	0.155
Dysuria	347	36.9	91	26.2	0.78 (0.58 to 1.05)	0.100	1.07 (0.74 to 1.54)	0.726
Other urinary symptoms^c^	252	26.8	80	31.7	1.69 (1.23 to 2.34)	0.001	1.21 (0.82 to 1.79)	0.341
Other symptoms^d^	178	18.9	29	16.3	0.82 (0.52 to 1.27)	0.372	0.67 (0.40 to 1.11)	0.116
Abdominal pain	160	17.0	58	36.3	0.94 (0.67 to 1.32)	0.709	1.10 (0.75 to 1.63)	0.622
Urinary urgency	143	15.2	39	27.3	0.88 (0.59 to 1.31)	0.526	0.81 (0.52 to 1.26)	0.345
Haematuria	128	13.6	53	41.4	1.93 (1.32 to 2.84)	0.001	2.07 (1.33 to 3.23)	0.001
Gynaecological symptoms	39	4.1	16	41.0	1.46 (0.78 to 2.75)	0.240	1.87 (0.95 to 3.68)	0.072
Bowel symptoms	19	2.0	8	42.1	2.92 (1.05 to 8.14)	0.040	3.31 (1.06 to 10.38)	0.04
Loss of appetite	12	1.3	4	33.3	1.26 (0.38 to 4.21)	0.710	0.43 (0.08 to 2.36)	0.332
Weight loss	6	0.6	5	83.3	12.76 (1.48 to 109.70)	0.020	24.54 (2.06 to 292.19)	0.011
Anaemia	3	0.3	3	100	Omitted^e^	—	Omitted^d^	—

a *Joint Wald test performed for all categorical variables.*

b *Missing data in each category.*

c *Other urinary symptoms include other storage and voiding symptoms, unspecified lower urinary tract symptoms, and descriptions about urine appearance.*

d *Other symptoms include systemic symptoms (such as fever and sweats), confusion, falls, malaise, and fatigue, for example.*

e *Omitted categories represent strata that were too small for regression to run. BMI = body mass index. IMD = Index of Multiple Deprivation. OR = odds ratio*.

In total, 268/940 (28.5%) patients had 356 referrals. Of these 356 referrals, the majority were to urology (*n* = 203, 57.0%), followed by ultrasound (*n* = 47, 13.2%) and gynaecology (*n* = 33, 9.3%). When examining the pattern of referral destinations by gender, nearly 80.0% (*n* = 116/149) of males were referred to urology, whereas urology (*n* = 87/207, 42.0%), gynaecology (*n* = 33/207, 15.9%), and ultrasound (*n* = 35/207, 16.9%) were the main referral routes for females (see Supplementary Table S3a).

#### Final diagnosis

There were 710/940 patients (75.5%) who had a recorded final diagnosis. Among all patients (including those without a diagnosis), the commonest final diagnosis was UTI (*n* = 456/940, 48.5%), followed by benign urological conditions (*n* = 124/940, 13.2%), other diagnosis (*n* = 97/940, 10.3%), and cancer (*n* = 33/940, 3.5%) (see Supplementary Table S3b). Cancer sites included prostate (*n* = 17), bladder (*n* = 7), lymphoma (*n* = 2), and upper tract urothelial cancer, myeloma, liver, pancreatic, ovarian, uterine, and testicular cancers (*n* = 1 each). When examining by gender, 376 (57.2%) female patients had a final diagnosis of UTI, with other diagnoses making up <5.0% each of the total number of female patients. In contrast, two main diagnoses — UTI (*n* = 80, 28.3%) and benign prostatic conditions (*n* = 62, 21.9%) made up about half of the diagnoses in males. Prostate cancer was the third most common diagnosis in males (*n* = 17, 6.0%). A similar proportion (about one-quarter) of males and females had no recorded diagnosis.

#### Diagnostic intervals

Of those referred, the PCI was the longest in patients with a final diagnosis of UTI (median 81.5 days). Patients with a final diagnosis of cancer had the longest (median 85 days) and those with presumed UTI the shortest (median 1.5 days) diagnostic intervals, respectively (see Supplementary Table S4).

#### Predictors of having a referral

Logistic regression showed higher odds of referral in male patients both crudely (odds ratio [OR] 2.65, 95% confidence interval [CI] = 1.97 to 3.58, *P*<0.001) and in the adjusted model (OR 3.03, 95% CI = 2.12 to 4.34, *P*<0.001) (Table 2). Patients with haematuria were more likely to be referred than those without (adjusted OR 2.07, 95% CI = 1.33 to 3.23, *P* = 0.001). There was a lack of association between individual urinary symptoms as presenting features (dysuria, frequency, or others) and likelihood of a referral. Patients who presented with bowel symptoms, weight loss or were underweight (BMI <18.5), and those in the least deprived quintile were also at increased odds of having a referral. Sensitivity analysis including an adjusted model containing the final diagnosis showed similar patterns of association between gender, deprivation, low BMI, and bowel symptoms, and odds of referral. The effect of haematuria was attenuated and weakened when final diagnosis was adjusted for (OR 1.59, 95% CI = 0.81 to 3.14, *P* = 0.179) (see Supplementary Table S5).

#### Patients with recurrent UTIs

In total, 238/940 (25.3%) patients in the sample had recurrent UTIs, and thus met NICE guidance for referral (the NICE-qualifying patient group): 201/238 (84.5%) of these were female. Of the 238 patients who qualified for NICE referral, 82 (34.5%) of them were referred; 62 (26.1%) were females, and 20 (8.4%) were males, with median PCIs of 35 and 2 days, respectively.

### Qualitative results

Fifteen patients (11 females and four males) between the ages of 45 and 84 years consented to an interview. Eight patients had UTI symptoms, five patients had haematuria, and two had both UTI symptoms and haematuria (see Supplementary Box S1).

#### Main findings

Quotations have been selected to best represent the four themes and each interviewee (identifier T) is described by their gender, age range, presenting features, final diagnosis, and investigations.

### The four key themes are:

- Missing information gathered about urinary symptoms;
- lack of clinical review in patients with recurrent UTIs;
- difficulty obtaining urine culture results; and
- patient autonomy (feeling able to challenge GPs).

#### A. Missing information gathered about urinary symptoms

Patients reported missing and inaccurate information being gathered during this part of the diagnostic process. One patient with recurrent UTIs presenting as urinary frequency remarked that:

*‘... nobody had actually said to me “Well how often are you going to the toilet?”.’*

(T9, female, aged 55–64 years, recurrent UTIs [RUTIs], ultrasound scan [USS], cystoscopy)

Female patients with recurrent UTIs also identified the lack of physical examinations despite having not responded to several courses of antibiotics:

*‘... they've never looked to see or asked why do you think you get so many* [UTIs] *… I don't have conversations with the doctor.’*

(T12, female, aged 75–84 years, RUTIs, no investigations)

#### B. Lack of clinical review in patients with recurrent UTIs

Female patients with recurrent UTIs described the lack of a follow-up clinician review following repeated attendances. They were sometimes prescribed multiple courses of antibiotics without seeing or speaking to a GP. The contacts that they did have with the practice were often with non-clinical staff such as receptionists, who were not always able to help with the management of their symptoms:

*‘… I assume they look at your notes and say “Well … you've had this* [antibiotic] *before, you've had that before, let's try that again”. And this went on for so many months and then I went back again.’*

(T3, female, aged 65–74 years, persistent cystitis, USS, cystoscopy)

*‘As I say, this last time, I had to have five lots* [of antibiotics] *and, in the end, I just gave up. You know, I thought, by the time I've taken some of five lots, it's helped itself* […] *It would be nice if when you phoned up and said you've got a water infection that the GP actually phones you to find out, instead of keep the receptionist doing it all … because then you could ask them the question, couldn't you? Why do I get so many of these and can you give me a continuity on antibiotics that I can live with and don't make me ill, you know?’*

(T12, female, aged 75–84 years, RUTIs, no investigations)

*‘No, I've had … Since about March, I've had, sort of, one* [antibiotic] *after the other. So those last lot of antibiotics … was the 15th lot I've had … one after the other.’*

(T14, female, aged 65–74 years, RUTIs, no investigations)

In contrast, one male patient with a possible UTI was referred for further investigations following their first presentation:

*‘I went to see them once and they then said, “Have the tests”.’*

(T6, male, aged 55–64 years, UTI, cystoscopy)

Partly because of the lack of re-assessment for persistent or recurrent symptoms, patients with UTIs often experienced a lack of, or delays in, referrals. In contrast, patients with haematuria often described guideline concordant management or prompt referrals by their clinicians. A patient with persistent non-visible haematuria described how her GP explicitly told her of the reason for her referral:

*‘… she reassured me and said “Look, they're very microscopic amounts and you're a very healthy, otherwise a very healthy person. But”, she said, “the guidelines state that I must do this now”.’*

(T7, female, aged 55–64 years, dysuria and non-visible haematuria, private computed tomography [CT], private cystoscopy)

Two male patients were referred on the fast-track referral route following their first presentations with haematuria:

*‘He said you have to do appointments for … one was ultrasound and the other one for … that's cystoscopy.’*

(T5, male, aged 45–54 years, visible haematuria, USS, cystoscopy, CT)

*‘I was referred to, for a camera for the bladder and the, the CT scan to check for kidney stones … it did come through very quick, because I went on the 28th* [to the GP] *and I got this appointment on the 1st.’*

(T11, male, aged 65–74 years, non-visible haematuria, USS, cystoscopy).

Another patient was offered further investigations following an episode of visible haematuria that responded to antibiotics, which he declined. Following a second episode of haematuria:

*‘I went to see the doctor again and we thought this time I ought to have a hospital test.’*

(T4, male, aged 75–84 years, visible haematuria, USS, cystoscopy).

#### C. Difficulty obtaining urine culture results

Patients described various process issues with getting the urine culture results. These included system issues, such as difficulty accessing the practice to get results, and the lack of follow-up and interpretation because of the result being delivered by a receptionist.

Furthermore, many patients reported the lack of a formal system for communication of test results, with results being only delivered if they were abnormal. The following quotes illustrate these issues:

*‘… because if you phone up and you'll get that you're thirteenth in the queue, well it puts you off phoning, doesn't it?’*

(T12, female, aged 75–84 years, RUTIs, no investigations)

*‘Well they say, the doctor will send this off, but never get any results back.’*

(T9, female, aged 55–64 years, RUTIs, USS, cystoscopy).

*‘They say, we'll send it to the hospital, but I never get any results back.’*

(T12, female, aged 75–84 years, RUTIs, no investigations)

*‘You'd only get a call if they did find something.’*

(T1, female, aged 75–84 years, non-visible haematuria, USS, cystoscopy)

*‘I phoned the doctor and it was the receptionist who told me that it was … there was just the one stone.’*

(T2, female, aged 55–64 years, non-visible haematuria, CT)

*‘… but the ball gets dropped a bit. And that's because you're dealing with a receptionist.’*

(T7, female, aged 55–64 years, dysuria and non-visible haematuria, private CT, private cystoscopy)

*‘So the second time when I needed different antibiotics they* [receptionist] *said to me “Oh, doctor's seen your blood results, yes, you've still got an infection, we need you to start on different antibiotics. A prescription will be ready” … ’*

(T9, female, aged 55–64 years, RUTIs, USS, cystoscopy)

#### D. Patient autonomy

Patients displayed different levels of patient autonomy with regards to their care. One described how she was reluctant to challenge her GP despite being given a shorter course of antibiotics than usual, and ended up having persistent symptoms:

*‘I suppose I'm quite a shy person and I should have said, why is it only* [a 3-day instead of a 7-day course of antibiotics?] … *but I didn't. I won't push myself forward at all.’*

(T15, female, aged 65–74 years, RUTIs, no investigations)

A few patients described trying to negotiate for a referral in the context of their GP's reluctance in referring them:

*‘Erm, I did create a fuss, I did say there's something wrong with me, I'm not right and I want to get to the bottom of it, whereas someone else might not be like that and they may have been fobbed off and … you know, to this day might not be dealt with.’*

(T9, female, aged 55–64 years, RUTIs, USS, cystoscopy)

### Mixed-methods synthesis

**Figure 1.**
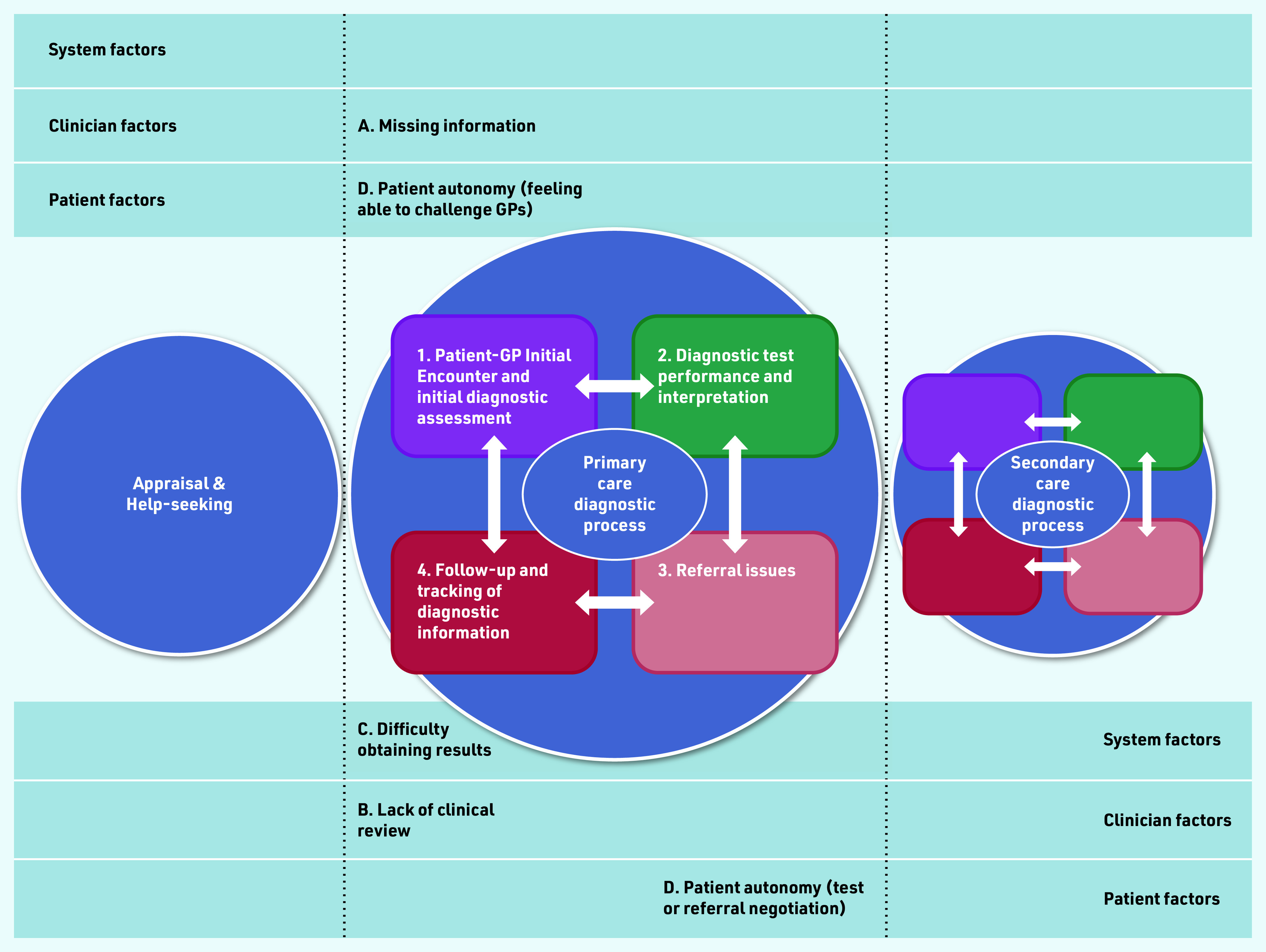
*Contributory themes (A–D) to potential missed diagnostic opportunities in the four diagnostic process dimensions (1–4) in primary care.*

The findings in the current study revealed that patients with presumed UTIs were at increased risk of MDOs, even if they qualified for referral under current NHS guidelines. Mapping these findings to the diagnostic process dimensions within the *Safer Dx* framework,^12^ potential barriers to timely diagnosis at the initial diagnostic assessment during patient–GP engagement, the diagnostic test performance and interpretation, referral, and follow-up and tracking of diagnostic information stages (Figure 1) were found in the current study.

Inadequate information gathering may lead to possible inaccurate diagnostic reasoning, and the failure to appreciate the changing risk of cancer in patients with recurrent and persistent symptoms. This may contribute to the observed lower referral odds in this group of patients and reduced guideline concordance in those with recurrent UTIs or UTI symptoms. Improving patient autonomy and confidence in challenging a GP when they did not agree with the management plan may reduce unnecessary delay in treatments, referral, and diagnosis.

Besides the initial diagnostic assessment, the follow-up stage of the diagnostic process was also prone to MDOs. Several clinician and system factors may contribute. Clinical assessment, follow-up, and referral decisions were less-guideline concordant in those with UTI symptoms, especially in females compared with males. All male interviewees were referred (or offered a referral) after their first presentation with a UTI (*n* = 1) or haematuria (*n* = 3).

In patients with haematuria, the gender inequity was less apparent, with clinicians generally making prompt referrals for both genders. This may explain the prolonged PCI seen in patients with recurrent UTIs, and the higher odds of referral in those with haematuria observed in the quantitative study. Finally, suboptimal communication of test results may hinder full diagnostic assessment and closing of the diagnostic loop, resulting in the failure to re-evaluate the clinical evidence in the evolving diagnostic process, and delays to subsequent referrals, if they occurred. This may explain the longer PCI seen in the relatively small proportion of patients with a final diagnosis of UTI who were referred, compared with that in the other diagnosis groups (see Supplementary Table S4).

Specific system barriers to timely results delivery include difficulties accessing the practice for results, communication of results by non-clinical staff, and the lack of proactive communication of urine culture results.

## DISCUSSION

### Summary

The findings of the current study suggest that patients with recurrent UTIs (especially females) who are at risk of cancer might be at increased risk of MDOs. Only one-third of females and half of males with recurrent UTIs in this sample were referred even if they met the NICE guidelines for further investigations, with males having a shorter PCI than females. Furthermore, the effect of haematuria was attenuated once the final diagnosis was adjusted for.

Given that about half of the patients had a final diagnosis of a UTI, it is likely that patients with haematuria attributed to UTIs were also less likely to be referred. Inadequate information gathering in the initial diagnostic assessment, system and clinician barriers in the follow-up of patients, and communication of results may contribute to MDOs.

### Strengths and limitations

Strengths of the current study include the prospective design, a relatively large sample size for case-note review, and the ability to capture delayed, missed, or non-events. This study used a mixed-methods approach to provide triangulated findings from different parts of the study to generate a richer understanding of MDOs and its contributors, regardless of the outcomes.

A limitation of the current study is that there was variation in the level of detail and completeness of the clinical data collected, partly because of different clinicians collecting the data and partly because of the complexity of some of the clinical cases. In particular, diagnosis data were missing in about 25% of the patients, which could represent the cases of patients where serious disease was ruled out but no diagnosis was found. Nevertheless, the missing data limited the ability to examine variations in outcomes by diagnosis group.

The current sample consisted of patients identified via coded symptoms and diagnosis that fitted the authors' inclusion criteria. This may result in over- or underestimation of the true odds of referral. However, given how common the included clinical features are in primary care, the effect of any potential bias is unlikely to be large. Further, the qualitative sample was relatively small and consisted mostly of females. Given that the most commonly diagnosed cancer was prostate cancer (which was expected) and no kidney cancer was diagnosed, comparison by cancer and gender was therefore restricted.

It was not possible to demonstrate objectively if harm was caused because of the lack of follow-up and delayed referrals in the current study. However, the qualitative interviews highlighted that the perceived delays resulted in negative patient experiences, which is increasingly important in the assessment of diagnostic performance in the broader context of value-based care including quality and risks.^23^ Finally, the results could have been strengthened if the authors had also interviewed GPs.

### Comparison with existing literature

The current findings supported existing evidence that patients with UTIs who were subsequently diagnosed with bladder or kidney cancer experienced a longer time to diagnosis,^24^ whether they first presented to a urologist or non-urologist.^25^ They were also less likely to be referred even if they met referral guidelines.^4^^,^^26^ The qualitative findings substantiated observations that females were more likely than males to receive ≥3 courses of treatments for UTIs before cancer diagnosis.^27^

Studies from the English primary care setting suggest that diagnostic errors account for the majority of avoidable significant harm,^28^ and that guideline discordance is not uncommon even for alarm symptoms.^29^ The qualitative findings in the current study emphasise problems in the patient–practitioner assessment; furthermore, system and clinician factors contribute to inadequate reappraisal of clinical information over time,^30^ and follow up of test results is prone to communication failures.^19^^–^^21^

### Implications for research and practice

The current study has several implications for practice, policy, and research. First, older females with UTI symptoms may be at increased risk of MDOs for bladder and kidney cancer. These findings highlight the need to establish the chronicity and pattern of urological symptoms during consultations. Current guidance from Public Health England suggests that the genitourinary syndrome of menopause should be considered, and that urine cultures should be sent before antibiotic prescriptions are given to older patients (aged >65 years) with suspected UTIs.^31^ However, evidence suggests that inadequate testing (such as use of urine culture at symptom recurrence)^32^ and the lack of pelvic examination^32^^,^^33^ are not uncommon in older females with urinary symptoms. Although system factors such as time constraints (the 10-min consultation norm in England)^5^ may contribute to suboptimal assessment, raising awareness of the patients at risk and the need to perform a complete assessment in some cases may overcome some of the cognitive biases faced by clinicians in older females with recurrent UTIs.

Next, patients reported that results were often given by non-clinical staff, which resulted in poor patient satisfaction as receptionists could not assist with clinical interpretation and follow-up activities. Previous successful systemwide interventions in the English primary care setting to improve test result communication and follow up have included supporting compliance with the Data Protection Act by receptionists, improving access to teleconsultations with GPs, training for receptionists in how to communicate potentially sensitive information, and providing a time slot for results communication by practice clinicians.^34^

Finally, ill-defined clinical criteria to diagnose UTIs,^35^ a lack of consensus on optimal antibiotic prescribing and symptom control of UTIs,^36^ and the lack of definitive guidelines on the management of recurrent UTIs^37^ might contribute to inconsistencies in the management of patients with persistent symptoms. There is an urgent need to refine and tighten the current NICE guidelines on recommendations for patients with recurrent UTIs, including the need to define ‘persistence’ and ‘recurrence’ of symptoms. To achieve this, further research is needed to improve the knowledge on positive predictive values of UTI for cancer, for single and repeated episodes, and in combination with other risk factors, so that better risk stratification strategies can be evaluated and implemented.

Nevertheless, electronic triggers used to flag up patients of interest in primary care have been successfully developed and tested in patients who were at risk of delayed or missed evaluation for possible cancer, including bladder cancer.^38^^–^^40^ These triggers could be a potential intervention to remind clinicians to review and reconsider management plans in patients who have had recent bouts of recurrent UTIs.

In conclusion, the current findings suggest that older female patients with UTIs present a clinical challenge to GPs. Improving clinician awareness of at-risk groups, and implementing system changes to improve test result communication may mitigate some MDOs. Future research is needed to inform the development and evaluation of risk-stratified approaches to improve management of patients with recurrent UTIs.

## Data

Data and code lists are available on request.
